# An integrated physics-guided machine learning approach for predicting asphalt concrete fracture parameters

**DOI:** 10.1038/s41598-025-32041-7

**Published:** 2026-03-02

**Authors:** Manzoor Elahi, Rawid Khan, Tufail Mabood, Muhammad Salman Khan, Awais Ahmed, Mahmood Ahmad, Zsolt Tóth

**Affiliations:** 1 Department of Civil Engineering, Main Campus, University of Engineering and Technology, Peshawar, 25000 Pakistan; 2 College of Civil and Transportation Engineering, Shenzhen University, Shenzhen, 518060 China; 3 Department of Built Environment, Oslo Metropolitan University, Oslo, Norway; 4 Institute of Energy Infrastructure, Universiti Tenaga Nasional, Kajang, 43000 Malaysia; 5 Department of Civil Engineering, University of Engineering and Technology Peshawar (Bannu Campus), Bannu, 28100 Pakistan; 6 Faculty of Wood Engineering and Creative Industries, University of Sopron, Sopron, Hungary

**Keywords:** Fracture energy, Asphalt mixtures, Physics-guided machine learning, Surrogate modeling, Single-edge notched beam, Surrogate fracture parameters, Engineering, Materials science, Mathematics and computing

## Abstract

Accurate prediction of fracture energy (G_f_) in asphalt mixtures is important for durable asphalt pavements designing. Traditional experimental approaches are reliable but need resources, whereas numerical simulations, such as finite element models (FEM), offer flexibility but needs accurate input parameters and calibration. Recent advances in machine learning offer rapid prediction capabilities; however, interpretability and physical relevance remain challenging in this regard. This study presents a hybrid framework that integrates experimental Single Edge Notch Beam (SENB) tests, finite element simulations, and machine learning models to predict fracture parameters for asphalt mixtures. Experimental testing quantified fracture energy, while FEM simulations replicated the fracture response numerically. Machine learning models, including Linear Regression, Gradient Boosting, and AdaBoost, were trained on mixture properties such as stability, flow, air voids, and Stiffness Modulus at 20 °C (ITSM20) to predict surrogate fracture energy. A novel, dimensionally consistent surrogate equation was proposed to link key mixture properties to fracture energy, validated against both experimental and numerical results. The surrogate model demonstrated best accuracy with a mean relative error compared to experimental data. This novel integrated approach, adopted in this study, provides a practical and physics-guided methodology for rapid and reliable prediction of fracture behavior in asphalt mixtures, bridging experimental observations, numerical simulations, and data-driven machine learning modeling, and offering insights for mixture optimization and pavement design.

## Introduction

The key understanding the fracture behavior of asphalt mixtures under mechanical loading, such as single edge notch beams is crucial for ensuring long-term pavement durability performance^1^. Over the past decades, researchers have employed both experimental and numerical techniques in their researches, to investigate the mechanical and fracture properties of asphalt concrete^2,3^. Tests in laboratories for material testing, such as the Single Edge Notch Beam (SENB) test, bending, and indirect tensile strength tests have been widely used to quantify fracture toughness, fracture energy, and load-deformation characteristics of asphalt mixtures^4–7^. These experimental testing methods provide a quick and direct measurement of asphalt performance but are often take a lot of time, need resources, man power and limited in the number of mixtures and conditions that can be tested^8–10^. Consequently, alternative approaches using numerical modeling, including finite element simulations, have gained traction, enabling researchers to predict fracture response under various loading conditions with reduced experimental effort^11–13^. Finite element methods are extensively used by the researchers in their research studies, specially, those implemented in software such as ABAQUS, have been applied in a very extended way, to check and simulate the stress distribution and crack propagation in notched asphalt beams, which offering a flexible framework for investigating complex material behavior such as asphalt by using Prony series coefficients or using the cohesive zone modeling. These numerical models, however, depend heavily on accurate input parameters and material characterization, which often introduces uncertainty into predictions^14–18^.

In parallel, the field of materials engineering has witnessed an interest, that is growing in machine learning (ML) and data-driven based methods to model material properties such as Asphalt^19–22^. ML models have the capability to capture the most complex, nonlinear relationships between mixture constituents, mechanical properties, and fracture response of asphalt, without relying solely on explicit physical formulations^22–24^. Few studies have applied algorithms in this advance filed simulation techniques, such as Linear Regression, Gradient Boosting, and AdaBoost to predict stiffness modulus, fatigue life, and rutting resistance in asphalt mixtures, demonstrating that data-driven approaches can complement experimental and numerical investigations in traditional laboratories. Yet, while these models provide rapid predictions, their interpretability and direct link to physical fracture parameters remain limited^24,25^. This has led to the necessity of hybrid physics-guided ML approaches, where surrogate fracture parameters derived from measurable mixture properties such as stability, flow, air voids, and stiffness serve as ML targets and bridging experimental observations and numerical predictions with predictive modeling using machine learning techniques in detail^26,27^.

Although there are substantial advancements, key research gaps are still present. Although there are substantial advancements, key research gaps are still present. First, few studies have integrated experimental, numerical, and machine learning approaches in a combined framework to systematically predict fracture energy and brittleness in asphalt mixtures^26–29^. While some published ML-based studies have examined conventional performance indicators such as stiffness or rutting resistance, while direct prediction of fracture energy for notched beams through machine learning especially when coupled with hybrid physics-guided surrogates remains largely unexplored^30,31^. Second, while surrogate fracture parameters have been conceptually proposed, there is a lack of practical formulations that are both dimensionally homogeneous and validated against experimental and FEM results^30,31^. Lastly, there is limited discussion on validating ML-based surrogate predictions against both experimental and numerical benchmarks to quantify their accuracy and robustness^32,33^.

This study addresses these gaps mentioned in the previous paragraph, by developing a hybrid framework that integrates experimental SENB tests (performed at university of engineering and technology Peshawar, Pakistan), FEM simulation with ABAQUS, and ML models to get the fracture toughness, use the predicted response in conjunction with the Python to further train on the dataset to predict surrogate fracture parameters in asphalt mixtures. Experimental work provides measured fracture response, FEM models simulate numerical behavior for the same specimens, and ML models including Linear Regression, Gradient Boosting, and AdaBoost predict surrogate fracture energy based on mixture constituents and mechanical properties. Overall, A novel surrogate fracture equation is proposed to link mixture properties (stability, flow, ITSM20, and beam dimension) to fracture energy, allowing a physically interpretable, dimensionally consistent prediction. This approach enables a rapid prediction of fracture energy without additional physical testing, followed by cross-validation against both experimental and numerical data, and exploration of parameter sensitivity to improve mixture design.

## Methodology

**Fig. 1:**
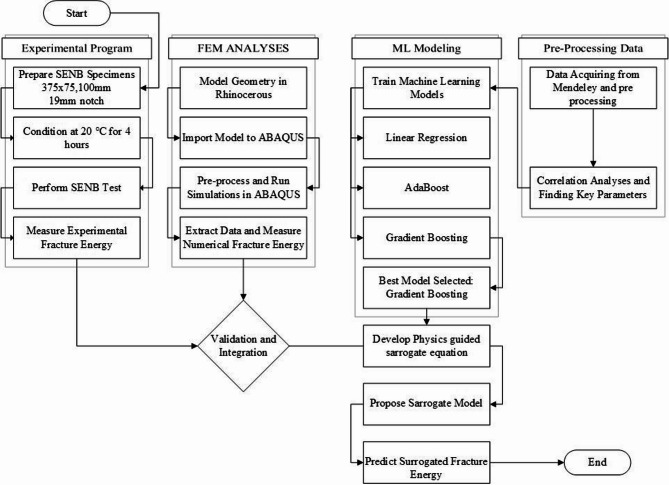
Hybrid framework for predicting fracture energy.

This study was conducted through an integrated, multi-stage methodology shown in Fig. 1 designed to develop a physics-guided machine learning framework for predicting fracture energy of the Single Edge Notch Beam. It started with an experimental program involving Single Edge Notch Beam (SENB) tests on asphalt mixtures to obtain benchmark fracture energy values. Concurrently, the finite element method (FEM) simulations were developed and validated against the experimental results of force displacement curve, to generate numerical fracture energy. A comprehensive dataset of mixture properties was subsequently utilized for machine learning modeling to perform correlation analysis and train multiple machine learning models including Linear Regression, Gradient Boosting, and AdaBoost. The core of the framework involved the development of a novelty based, dimensionally consistent surrogate equation that synthesizes key mixture properties. At the final stages, the predictions from the surrogate model were validated against both experimental and numerical benchmarks to ensure accuracy and physical relevance. To clearly articulate this progression, Sect. 3 is structured to mirror the methodological sequence. Section 3.1 presents the experimental procedures and key fracture measurements that serve as the foundational reference. Section 3.2 then introduces the FEM model development and its validation using experimental force–displacement curves. Section 3.3 focuses on the derivation of the surrogate fracture equation, highlighting its dimensional consistency and its role in linking physical parameters to fracture energy. Finally, Sect. 3.4 describes the machine learning models trained using both raw mixture properties and the surrogate-derived parameters, followed by a comprehensive evaluation against experimental and numerical benchmarks.

### Experimental program

**Fig. 2:**
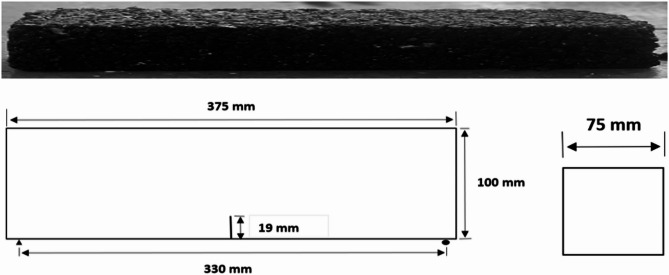
Single edge notch beam dimensions.

The experimental program was designed to obtained precise data on the fracture behavior of asphalt concrete, which subsequently provided the foundation for finite element analyses and machine learning prediction. Single Edge Notch Beam (SENB) samples were made in a standard rectangular shape with dimensions of 375 mm in length, 75 mm in width, and 100 mm in height. To ensure a controlled crack initiation path, a middle notch of 19 mm depth was made at the center of each sample, as shown in Fig. 2. The aggregates used in this study were obtained from the KARKON Plant located near the Motorway Toll Plaza, with the source material originating from the Margalla region. These aggregates were selected to meet the National Highway Authority (NHA) Class B gradation specifications. Asphalt Cement (Bitumen) of grade 60–70 was procured from Attock Refinery Limited, Rawalpindi.

**Fig. 3:**
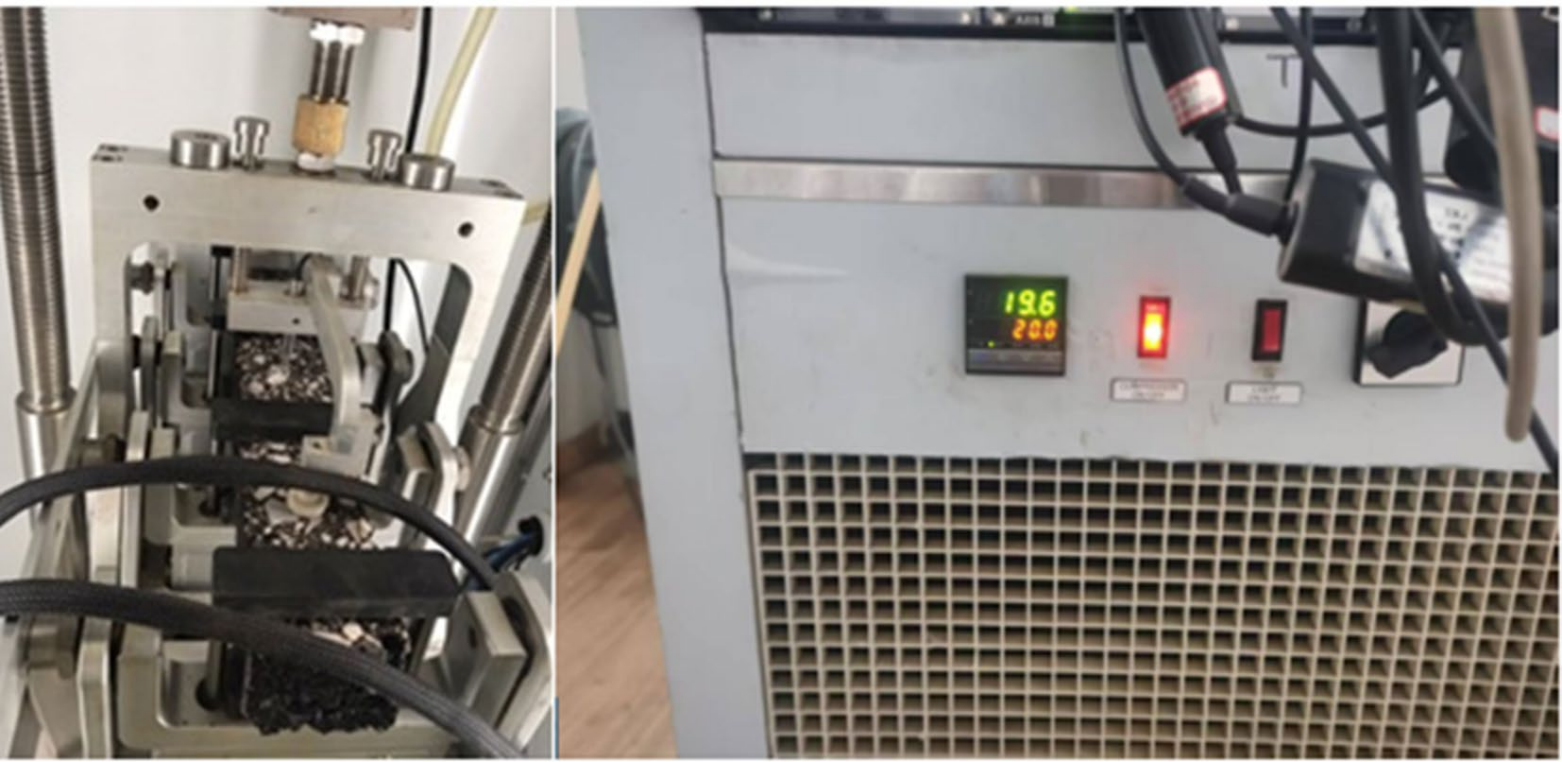
Single edge notch beam testing.

To study how temperature affects cracking, the samples were kept in in a temperature-controlled chamber for conditioning. Before testing, each beam was kept at the set temperature for 4 h to ensure uniform temperature. The main testing temperature was 20 °C, and tests were performed with a third-point bending setup using a displacement-controlled loading system, as shown in Fig. 3. For each bitumen content level, three specimens were prepared to ensure consistency and allow for reliable averaging of results. A total of five bitumen content levels were tested, resulting in 15 specimens overall (5 bitumen contents × 3 specimens per content level). During testing, the applied load and corresponding mid-span displacement were continuously recorded until complete failure of the specimen. The load–displacement data were then processed to compute fracture energy (Gf), which represents the energy absorbed by the mixture per unit crack surface area. This metric provided a quantitative measure of the mixture’s resistance to crack initiation and propagation, forming the basis for subsequent finite element validation and the development of surrogate machine learning models.

### FEM analyses

For the Finite Element Method (FEM) component of the study, simulations were conducted exclusively at 20 °C to correlate with the experimental data at that temperature. The viscoelastic behavior of the asphalt was modeled using Prony series coefficients. All the inputs and details are tabulated in Table 1.

**Table 1:** Material properties and constants used in ABAQUS model.

Parameter type	Property/coefficient	Value	Unit	Temperature (K/°C)	Notes
Material name	–	Python Generated Asphalt Mat	–	–	Defined in input file
Density	ρ	2.4 × 10⁻⁹	tonne/mm³ (≈ 2400 kg/m³)	293 K (20 °C)	Standard asphalt mixture density
Elastic modulus	E	1000	MPa	293 K (20 °C)	Long-term modulus
Poisson’s ratio	ν	0.3	–	293 K (20 °C)	Typical for asphalt
Viscoelastic (Prony series)	g₁	0.022834	–	–	Time-domain coefficients
	k₁	0.000969	–	–	
	τ₁	0.02007	s	–	
	g₂	0.022945	–	–	
	k₂	0.002201	–	–	
	τ₂	0.045604	s	–	
	g₃	0.023056	–	–	
	k₃	0.003433	–	–	
	τ₃	0.071138	s	–	
	g₄	0.023167	–	–	
	k₄	0.004666	–	–	
	τ₄	0.096673	s	–	
	g₅	0.023278	–	–	
	k₅	0.005898	–	–	
	τ₅	0.122207	s	–	
	g₆	0.023389	–	–	
	k₆	0.007131	–	–	
	τ₆	0.147741	s	–	
	g₇	0.0235	–	–	
	k₇	0.008363	–	–	
	τ₇	0.173275	s	–	
	g₈	0.023612	–	–	
	k₈	0.009595	–	–	
	τ₈	0.198809	s	–	
	g₉	0.023723	–	–	
	k₉	0.010828	–	–	
	τ₉	0.224344	s	–	
	g₁₀	0.023834	–	–	
	k₁₀	0.01206	–	–	
	τ₁₀	0.249878	s	–	
Physical constants	Absolute zero reference	−273.15	°C	–	Defined in input file

**Fig. 4:**
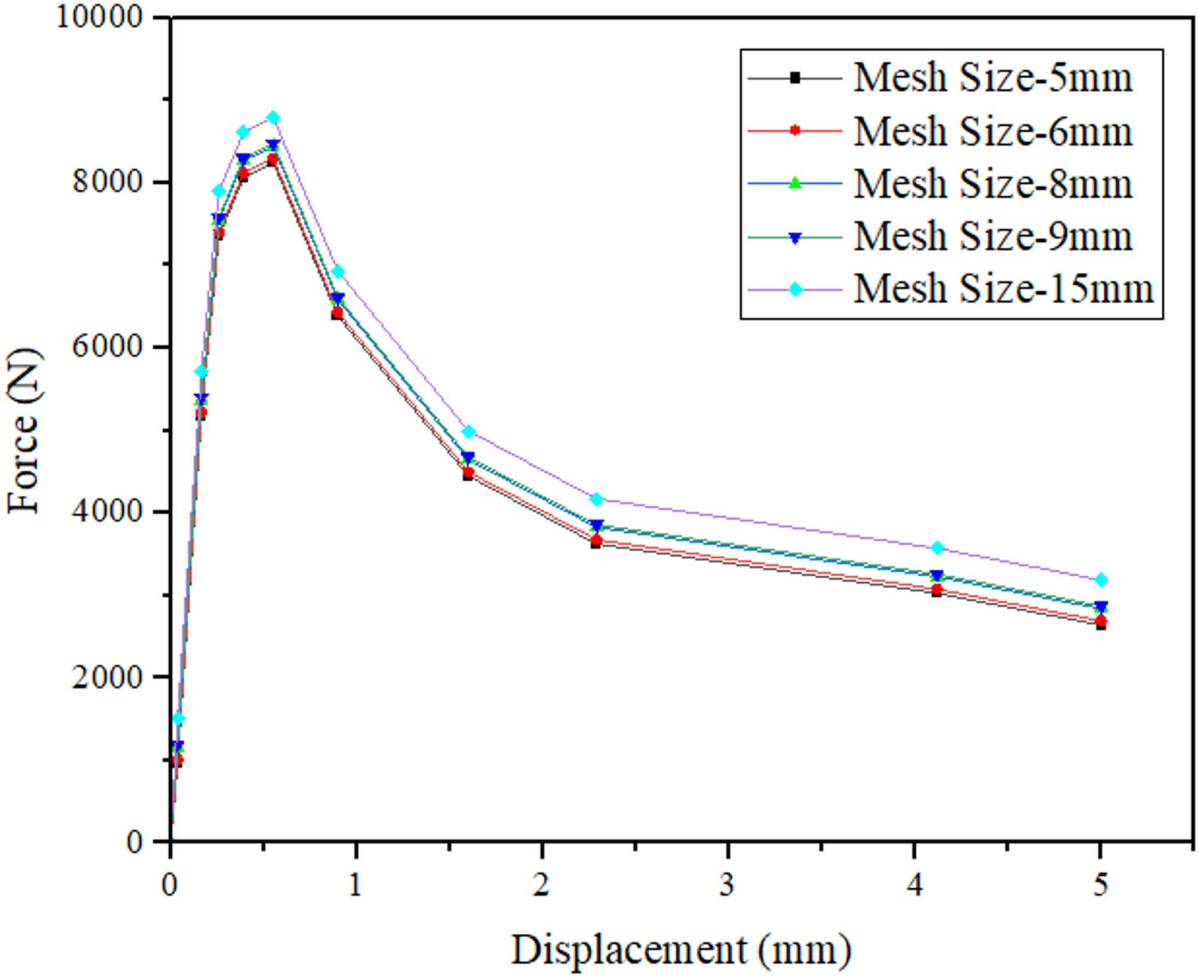
Force–displacement curve comparison for different mesh densities (Mesh sensitivity study showing overlapping curves for 5 mm and 6 mm meshes, confirming mesh-independent behavior).

**Fig. 5:**
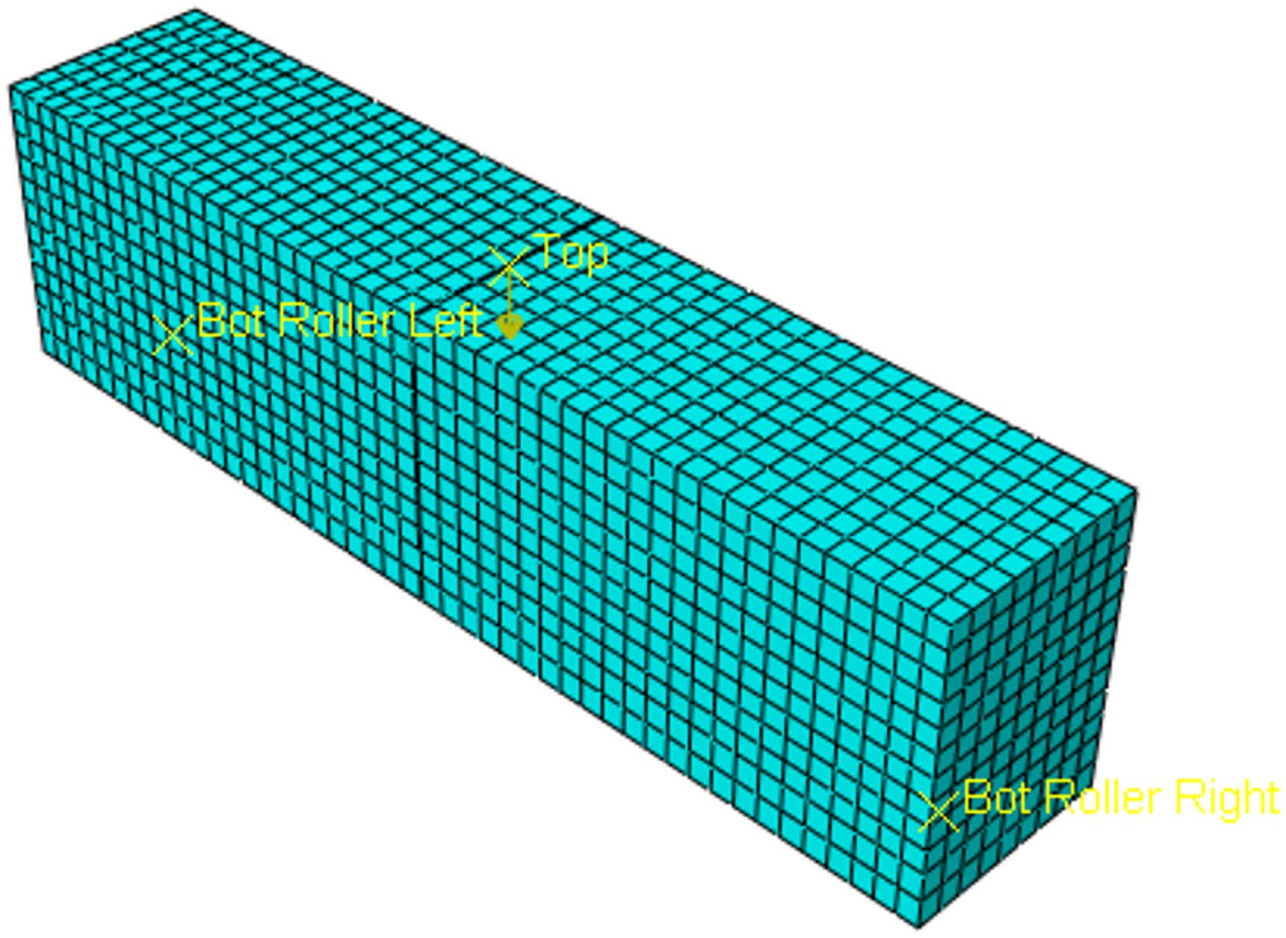
Meshed ABAQUS model.

The beam geometry, including the single-edge notch, was first created in Rhinoceros software and subsequently imported into Abaqus/CAE. Within Abaqus, the material properties were assigned, and boundary conditions were established to replicate a third-point loading test setup. A displacement-controlled analysis was performed by applying a 5 mm displacement at the loading points. A comprehensive mesh sensitivity analysis was conducted prior to the final simulations in which five global seed sizes, that are 15 mm, 9 mm, 8 mm, 6 mm, and 5 mm were tested. The comparison was based on the force–displacement curves obtained from the SENB simulations. The corresponding curves to the 5 mm and 6 mm mesh, as shown in Fig. 4, were nearly identical in both peak load and overall curve shape, indicating mesh-independent behavior beyond this refinement level. Consequently, a 5 mm global mesh size was selected for all subsequent analyses as it provided sufficient accuracy with an optimal computational cost. A meshed model is shown in Fig. 5 for visualization purpose.

Following the FEM simulations, the focus shifted to the fracture mechanics and machine learning (ML) phase of the study. The force-displacement data and the evolution of stress fields from the Abaqus models were extracted to calculate key fracture parameters, specifically the fracture energy. This computationally derived fracture energy was subsequently utilized for two primary purposes, that are, validation through comparison with experimentally obtained values, and to serve as the target variable for developing a surrogate model via machine learning for the data set downloaded from Mendeley website (https://data.mendeley.com/datasets/yb4brz3sdx/3) .

### Interpretation of correlation matrix (w.r.t ITSM20)

**Fig. 6:**
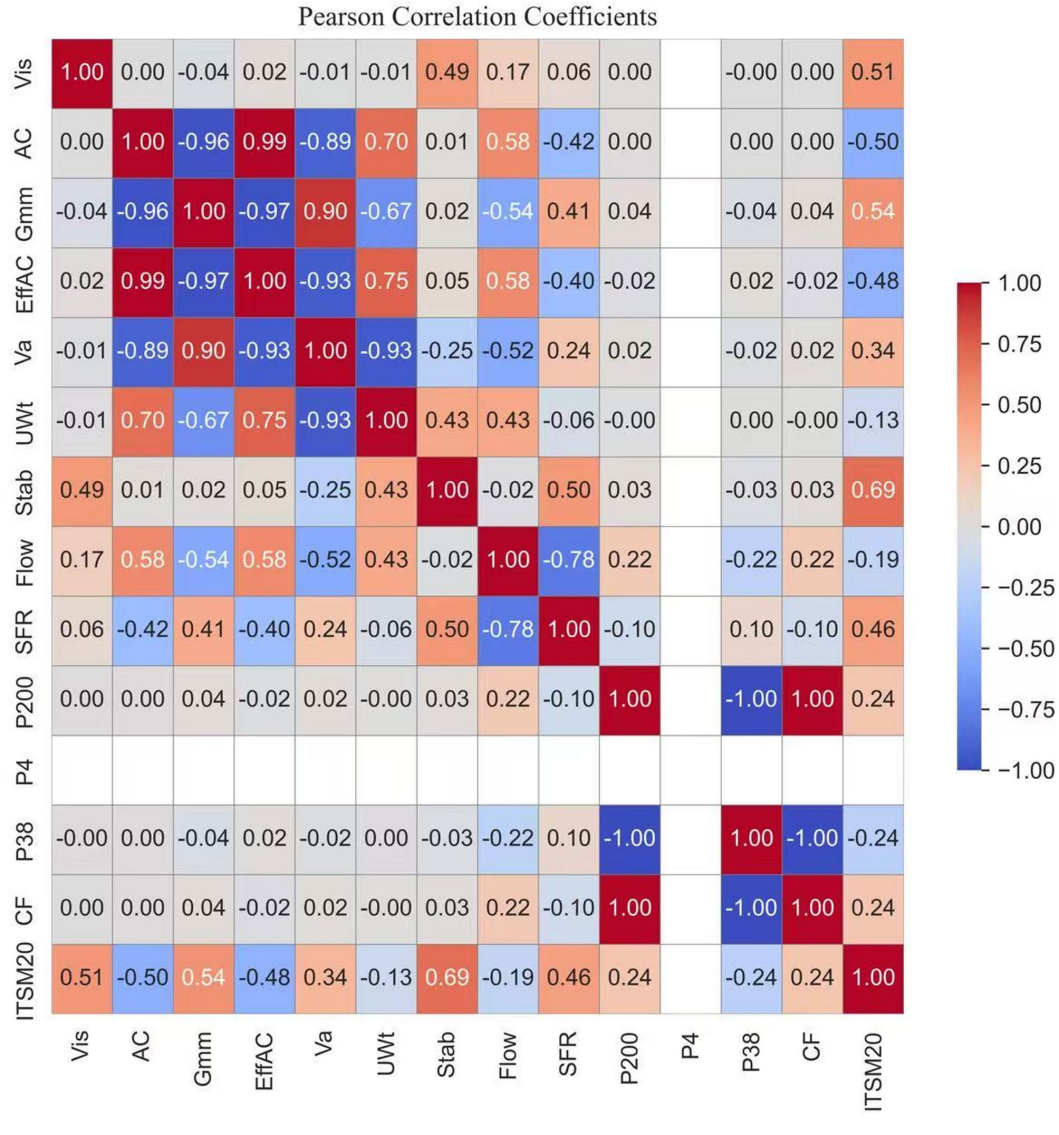
Pearson correlation.

Before starting machine learning modeling, the correlation analysis was performed which showed that ITSM20 is strongly related to stability which confirms the relationship between stiffness and load bearing capacity. Positive correlations with maximum specific gravity and viscosity suggest that dense aggregate packing and binder stiffness enhance mixture rigidity in resisting the load, which was the key parameter in the experimental program as well. Stability to flow ratio also increases with stiffness which points to higher brittleness in stiffer mixtures, which is opposite of experimental program. In contrast asphalt content and effective asphalt content are negatively correlated with ITSM20 reflecting the tradeoff between binder richness and stiffness where richer mixtures are softer but more ductile, as observed in experimental program. Flow also shows a weak negative link with stiffness supporting the idea that more deformable mixtures absorb more energy but resist less load. Overall, the results confirm that stiffness and stability govern toughness while binder content influences fracture energy and ductility. All correlation coefficients are shown in Fig. 6. Table 2 presents the Pearson correlation matrix p-values showing the relationships among key asphalt mixture parameters and the Indirect Tensile Stiffness Modulus at 20 °C (ITSM20).

**Table 2:** Pearson correlations P-values.

	Vis	AC	Gmm	EffAC	Va	UWt	Stab	Flow	SFR	P200	P4	P38	CF	ITSM20
Vis	0.00	1.00	0.68	0.79	0.88	0.94	0.00	0.05	0.47	1.00		1.00	1.00	0.00
AC	1.00	0.00	0.00	0.00	0.00	0.00	0.90	0.00	0.00	1.00		1.00	1.00	0.00
Gmm	0.68	0.00	0.00	0.00	0.00	0.00	0.85	0.00	0.00	0.68		0.68	0.68	0.00
EffAC	0.79	0.00	0.00	0.00	0.00	0.00	0.57	0.00	0.00	0.81		0.81	0.81	0.00
Va	0.88	0.00	0.00	0.00	0.00	0.00	0.00	0.00	0.00	0.83		0.83	0.83	0.00
UWt	0.94	0.00	0.00	0.00	0.00	0.00	0.00	0.00	0.48	0.96		0.96	0.96	0.14
Stab	0.00	0.90	0.85	0.57	0.00	0.00	0.00	0.84	0.00	0.74		0.74	0.74	0.00
Flow	0.05	0.00	0.00	0.00	0.00	0.00	0.84	0.00	0.00	0.01		0.01	0.01	0.03
SFR	0.47	0.00	0.00	0.00	0.00	0.48	0.00	0.00	0.00	0.27		0.27	0.27	0.00
P200	1.00	1.00	0.68	0.81	0.83	0.96	0.74	0.01	0.27	0.00		0.00	0.00	0.00
P4														
P38	1.00	1.00	0.68	0.81	0.83	0.96	0.74	0.01	0.27	0.00		0.00	0.00	0.00
CF	1.00	1.00	0.68	0.81	0.83	0.96	0.74	0.01	0.27	0.00		0.00	0.00	0.00
ITSM20	0.00	0.00	0.00	0.00	0.00	0.14	0.00	0.03	0.00	0.00		0.00	0.00	0.00

### Machine learning framework

After performing correlation analyses, three machine learning algorithms were implemented to predict fracture related responses from the asphalt mixture dataset, details of which are tabulated in Table 3. In the machine learning framework, the predictive models were trained using a comprehensive set of mixture design, physical, mechanical, and gradation parameters as input features, namely viscosity (Vis), asphalt content (AC), maximum theoretical specific gravity (Gmm), effective asphalt content (EffAC), air voids (Va), unit weight (UWt), stability (Stab), flow value (Flow), stability-to-flow ratio (SFR), percent passing sieve #200 (P200), percent retained on sieve #4 (P4), percent retained on sieve #3/8 (P38), and the coarse-to-fine ratio (CF). These inputs collectively capture the effects of binder rheology, mixture volumetrics, and aggregate structure on mechanical response. The target output for all machine learning models was the Indirect Tensile Stiffness Modulus at 20 °C (ITSM20), which was selected as the primary response variable due to its relevance in characterizing mixture stiffness under service conditions. No models were trained using data at 30 °C. Three algorithms such as Linear Regression, Gradient Boosting, and AdaBoost were evaluated using this same input–output structure across multiple cross-validation folds. Gradient Boosting was applied using scikit learn with 100 trees, a learning rate of 0.1, maximum depth of 3 for individual trees, minimum split size of 2, and a full training fraction to ensure replicable performance. AdaBoost was employed with decision tree estimators as the base learner, 50 estimators in total, a learning rate of 1.0, and linear regression loss for continuous target prediction. A standard Linear Regression model without regularization was also used as a baseline due to its interpretability and direct mapping of input parameters to output response. These models were trained under identical conditions and later validated using k fold cross validation to assess robustness and generalization capability.

**Table 3:** Machine learning models and their parameter configurations.

Model	Parameter	Value/setting	Description	Data Partitioning
Gradient Boosting	Implementation	scikit-learn	Python library used for model development	10-fold cross-validation (90% train / 10% test per fold)
	Number of trees	100	Total estimators in the ensemble	
	Learning rate	0.1	Step size controlling contribution of each tree	
	Maximum depth	3	Limits complexity of individual trees	
	Minimum samples split	2	Minimum number of samples required to split a node	
	Training fraction	1.0 (full dataset)	Ensures replicable performance	
	Validation method	k-fold cross-validation	Evaluates model robustness and generalization	
AdaBoost	Implementation	scikit-learn	Python library used for model development	10-fold cross-validation (90% train / 10% test per fold)
	Base estimator	Decision Tree Regressor	Weak learner for boosting	
	Number of estimators	50	Total boosting iterations	
	Learning rate	1	Weight applied to each estimator	
	Loss function	Linear regression loss	Appropriate for continuous targets	
	Validation method	k-fold cross-validation	Used for model robustness assessment	
Linear Regression	Implementation	scikit-learn	Python library used for model development	10-fold cross-validation (90% train / 10% test per fold)
	Regularization	None	Standard linear regression without penalty	
	Model type	Ordinary Least Squares (OLS)	Direct mapping between inputs and outputs	
	Validation method	k-fold cross-validation	Consistent with other models for comparison	

### Cross validation procedure and error analysis

**Fig. 7:**
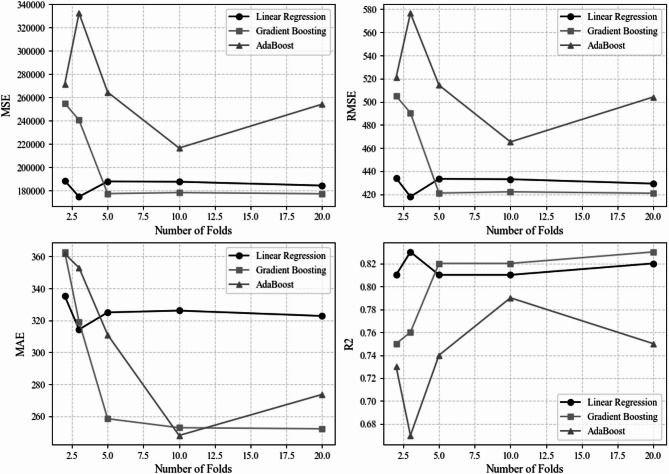
Error analysis.

Following The correlation analyses, A k fold cross validation procedure was applied to check the effectiveness of the machine learning models. Different fold numbers including 2, 3, 5, 10, and 20 were tested in all the chosen ML algorithms as shown in Table 4, in order to examine the effect of fold selection on the stability of the performance metrics. This approach ensured that the models were assessed under varying validation schemes and reduced the risk of overfitting to a specific data split. Figure 7 illustrates a cross-validation-based error analysis of three machine learning models across four key metrics (MSE, RMSE, MAE, and R^2^) as the number of folds varies from 2.5 to 20. Both Linear Regression and Gradient Boosting were superior models, with Gradient Boosting providing a slight edge in minimizing absolute error at higher fold counts, while AdaBoost was the least suitable choice. The 10-fold cross-validation was finally adopted, as it provided the most consistent R² and error metrics across models, ensuring a balanced and reliable evaluation framework.

**Table 4:** K-Fold study performed for all ML models.

Model	MSE	RMSE	MAE	MAPE	R2	Number of folds
Linear Regression	188415.38	434.07	335.28	8.64	0.81	2
Gradient Boosting	255052.04	505.03	362.82	9.81	0.75	2
AdaBoost	271540.33	521.10	361.69	9.95	0.73	2
Linear Regression	174700.57	417.97	314.34	8.05	0.83	3
Gradient Boosting	240544.01	490.45	319.16	8.63	0.76	3
AdaBoost	332477.19	576.61	352.92	9.45	0.67	3
Linear Regression	187830.23	433.39	325.02	8.34	0.81	5
Gradient Boosting	177329.34	421.10	258.55	6.70	0.82	5
AdaBoost	264542.67	514.34	311.05	7.98	0.74	5
Linear Regression	187530.01	433.05	326.15	8.33	0.81	10
Gradient Boosting	178226.22	422.17	252.92	6.72	0.82	10
AdaBoost	216532.59	465.33	248.08	6.60	0.79	10
Linear Regression	184273.87	429.27	322.80	8.29	0.82	20
Gradient Boosting	177208.49	420.96	252.26	6.74	0.83	20
AdaBoost	254190.28	504.17	273.72	7.22	0.75	20

### Surrogate fracture parameters

After selection of machine learning algorithms, surrogate fracture parameters were defined based on key mixture properties from the dataset. These parameters integrate stability, flow, and stiffness modulus to represent fracture energy in a physically meaningful and dimensionally consistent manner. A characteristic beam dimension (k) is incorporated as a scaling factor to relate the surrogate parameter to single-edge notch beam mechanics based on pure novelty. This formulation allows estimation of fracture energy for each dataset sample without performing full experimental tests thus bridging the gap between observed material behavior and predictive modeling with machine learning modeling. The surrogate parameters in this study, serve as target responses for the machine learning models, ensuring that predictions remain grounded in interpretable material behavior observed in experimental program, while enabling rapid, reliable assessment of fracture performance across different asphalt mixtures. This approach adopted in this study, provides a novel, hybrid framework that combines experimental work performed, numerical simulation done, and data-driven insights for practical pavement design and mixture optimization.

## Discussion

### Load–displacement behavior of FEM and experimental result

**Fig. 8:**
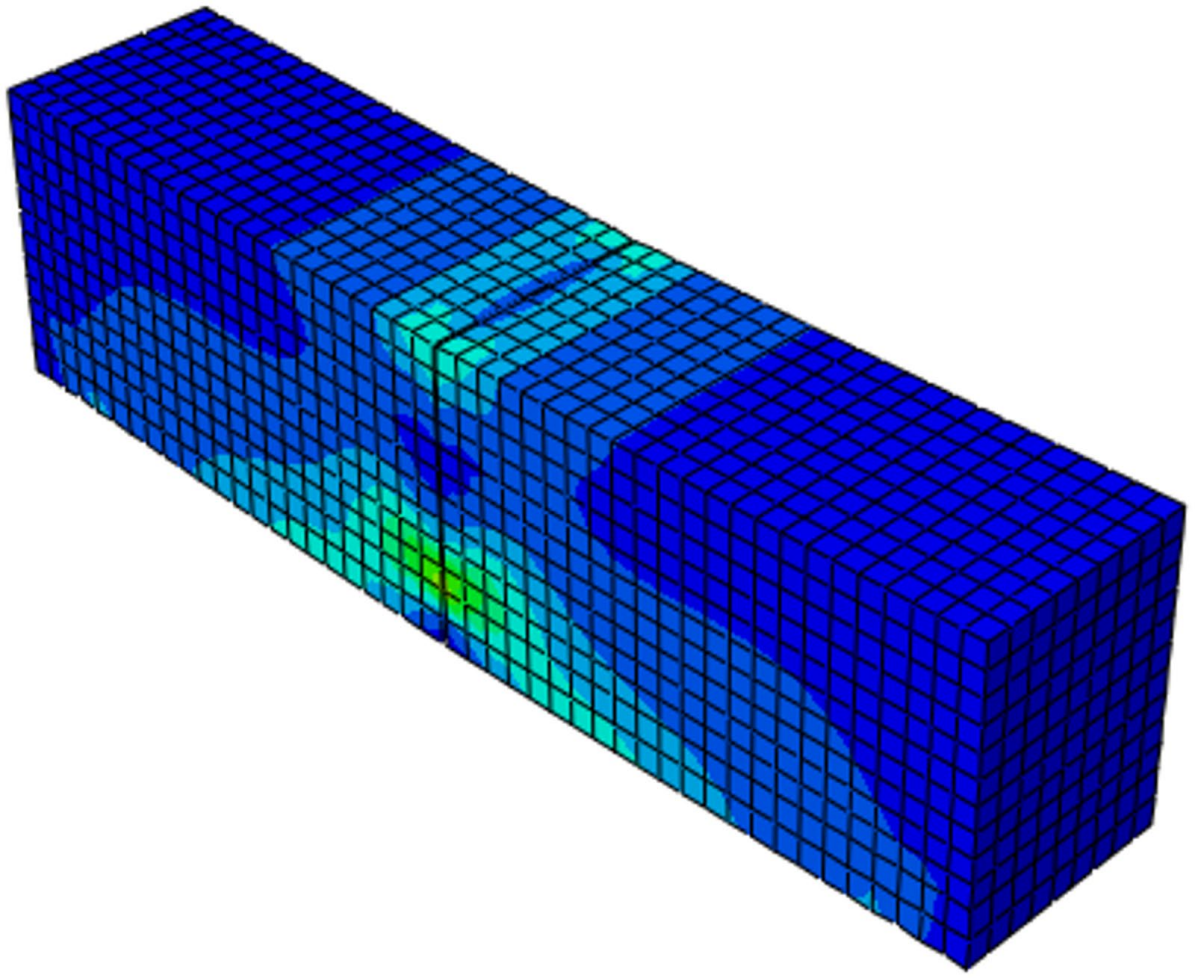
Deformed shape of asphalt (Contours showing the strain).

**Fig. 9:**
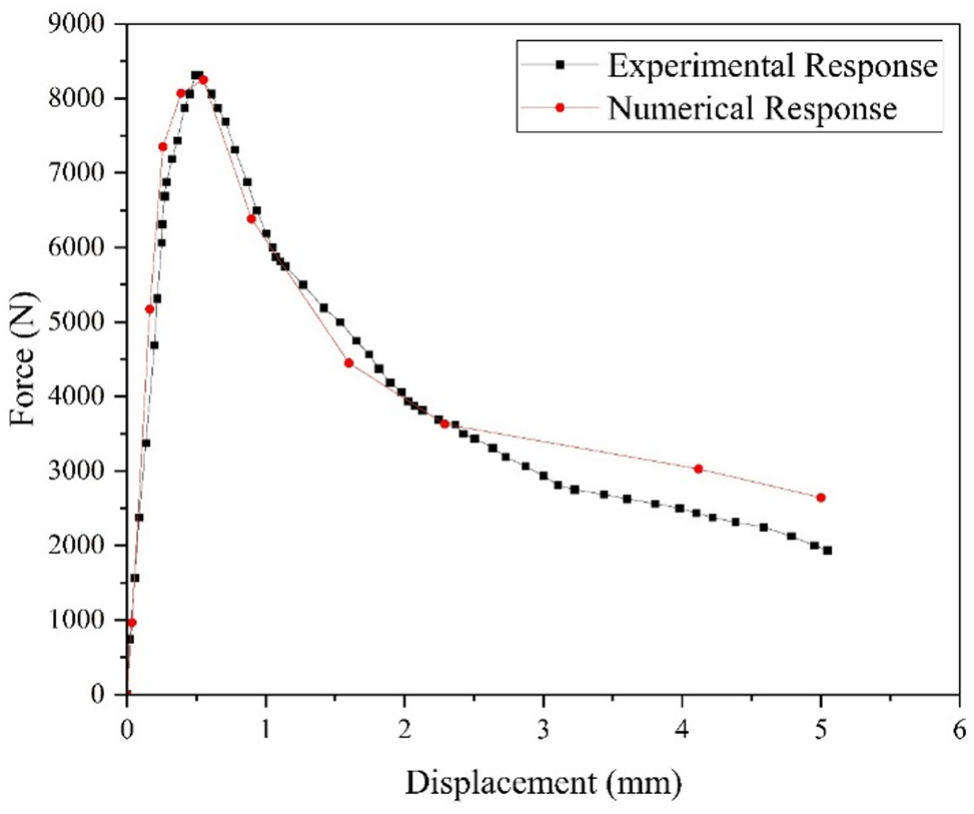
Comparison of actual vs. numerical response of FD Curve.

After completing the experimental program and finite element analyses, the comparison between the finite element model (shown in Fig. 5) and the experimental single edge notched beam (Shown in Fig. 3) results showed good agreement in terms of overall load–displacement behavior as shown in Fig. 9. Both approaches captured the initial linear response followed by gradual softening after peak load, which is characteristic of quasi brittle fracture in asphalt mixtures. The FEM predicted a peak load of around 8.3 kN occurring at a mid-span displacement close to 0.5 mm, which closely matched the experimental measurements. The failure mode is shown in Fig. 8 which matches the experimental failure observed in Fig. 3.

After the peak, the load carrying capacity gradually decreased with increasing displacement gradually through solver controls, and the simulation reproduced the tail of the curve with reasonable accuracy, although it exhibited slightly stiffer post peak behavior compared to the experimental beam as shown in Fig. 9. This difference may be attributed to material heterogeneity and micro cracking in the laboratory specimens that are not fully represented in the numerical model but overall, the close alignment of peak load, stiffness, and softening trend confirmed the reliability of the FEM results.

### Experimental program insights

**Fig. 10:**
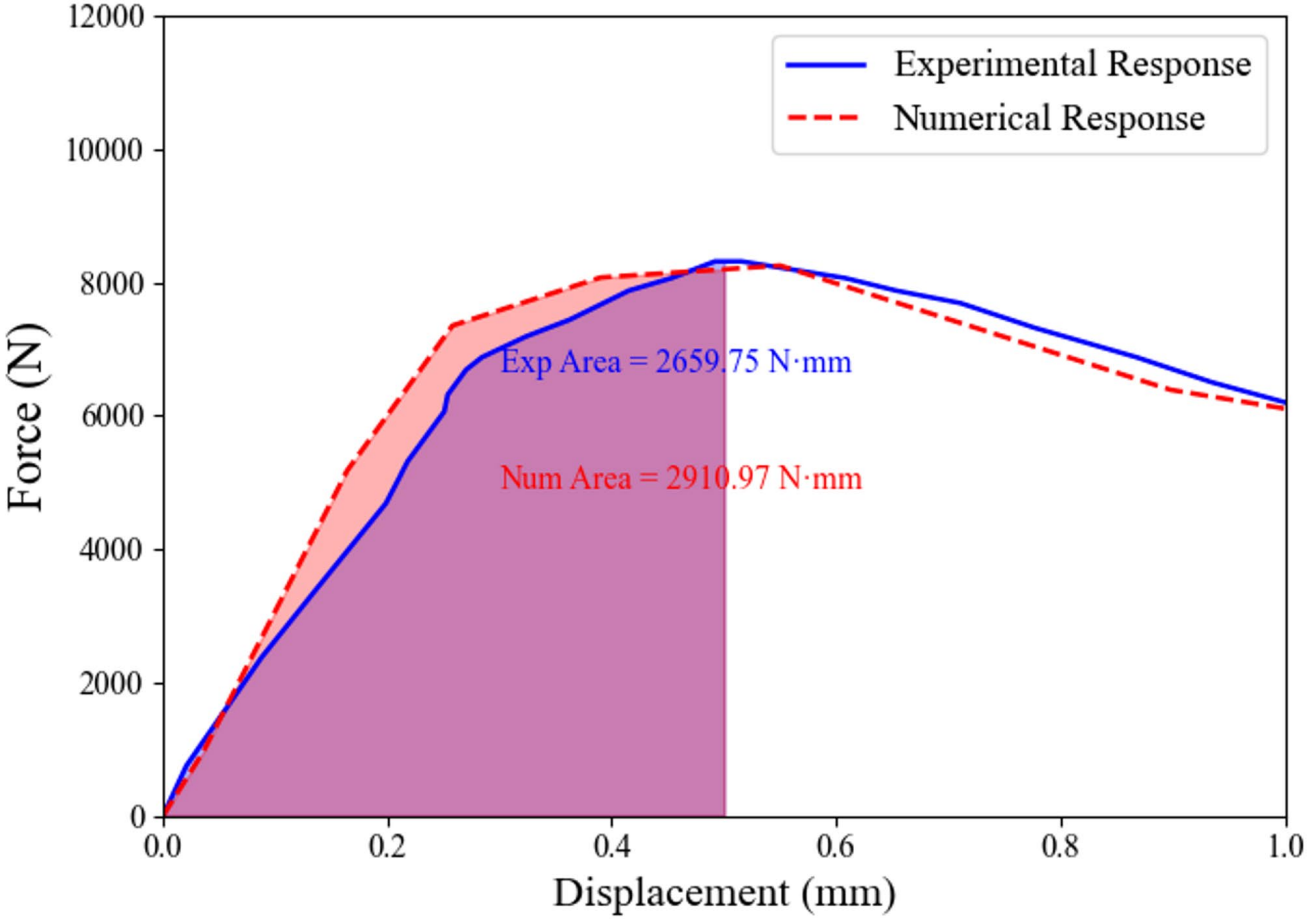
Fracture energy comparison derived from experimental and numerical load–displacement data.

The experimental program provided clear evidence of how asphalt mixture composition influences fracture behavior observed in the testing evaluation. Mixtures with higher stability values exhibited steeper load displacement slopes, reflecting increased stiffness but also a tendency toward brittle failure once the peak capacity of the single notch beam is obtained. Flow values revealed the role of asphalt content, where higher binder levels led to greater deformation capacity and energy absorption, but reduced overall stiffness as shown in Fig. 10. The balance between stability and flow highlighted the classic trade-off between strength and ductility in asphalt systems as visible by the work done highlighted in the Fig. 10. These findings validate the use of surrogate fracture parameters and form the basis for integrating experimental evidence with the FEM predictions and the machine learning framework.

### Correlation matrix interpretation

The correlation matrix in Fig. 6, highlighted the dominant role of stiffness modulus at 20 °C in relation to mixture properties. A strong positive correlation with stability (0.695) confirmed that mixtures capable of carrying higher loads also exhibited greater stiffness. Moderate positive links with maximum theoretical specific gravity (0.539) and viscosity (0.513) suggested that dense aggregate packing and stiffer binders contribute to higher modulus values. But on the other hand, asphalt content (–0.500) and effective asphalt content (–0.481) showed negative correlations, reflecting the well-known trade off where richer binder mixtures are softer but more ductile. The stability to flow ratio (0.462) also increased with stiffness, indicating more brittle behavior in stiffer mixtures. Air voids (0.342) and fines content (0.241 with P200) properly showed a weaker positive association, while flow (–0.191) and unit weight (–0.127) showed weak negative relationships. Overall, the correlations reinforced that stiffness and stability govern load resistance, whereas binder content and flow control the energy absorption capacity of asphalt mixtures.

### Surrogate fracture parameters and physical justification

The novelty based surrogate fracture energy ($\:{G}_{f}$) was formulated, through Python, to capture the fracture behavior of asphalt mixtures using readily same available material properties from the dataset, which were used in the machine learning algorithms. Key parameters are mixture stability, flow, and stiffness modulus were selected based on their established influence on load-bearing capacity, brittleness, and energy dissipation. The surrogate formulation is shown in Eq. 1 combines these factors to approximate fracture energy in a dimensionally consistent manner, where the characteristic beam dimension is also included as a physical scaling factor.

1 $$\:\begin{array}{c}{G}_{f}=k*\left\{Stab\left(1+D\right)+Flow\left(ITSM20\right)\right\}\end{array}$$

Where: $\:{G}_{f}$ = surrogate fracture energy (N·mm], $\:k$ = scaling constant (dimensionless), $\:Stab$ = mixture stability [N], $\:D$ = characteristic beam dimension [mm], $\:Flow$ = mixture flow [mm], $\:ITSM20$ = stiffness modulus at 20 °C [N/mm²].

**Fig. 11:**
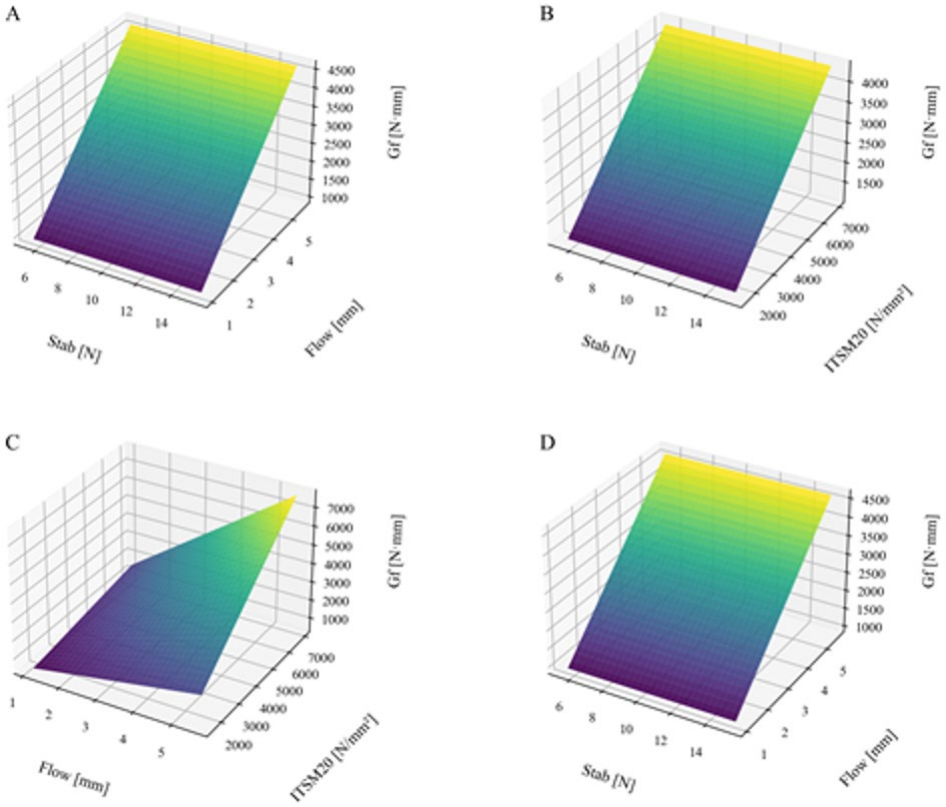
Variation of surrogate fracture energy with key mixture parameters across the dataset.

This approach is physically justified: higher stability and stiffness increase mixture rigidity and fracture resistance, while flow captures deformability under loading. Figure 11 presents four 3D surface plots (A–D) illustrating the variation of surrogate fracture energy with key mixture parameters across the dataset, enabling visualization of the combined effects of stability, flow, and stiffness modulus.

**Table 5:** Surrogate fracture energy values computed using only Stab, Flow, ITSM20.

Stab	Flow	ITSM20	Gf
9.101029	2.3622	4023	1923.128
10.79594	2.2352	4338	1965.678
7.526036	2.2098	3756	1678.38
5.461002	2.8194	2319	1321.639
8.936968	2.1844	4506	1990.36
12.22918	2.0828	5162	2179.835
11.81969	3.175	4412	2832.765
8.919073	1.8542	3708	1396.22
7.755722	2.1336	3323	1436.812

Table 5 contains the surrogate fracture energy values computed using only Stab, Flow, ITSM20, and the characteristic beam dimension D, along with the corresponding input ranges for these parameters, clearly documenting the domain used for Gf estimation. With these parameters tabulated in Table 5, the surrogate parameter provides an interpretable estimate of fracture energy that aligns with single-edge notch beam mechanics and bridges experimental and computational observations. The proposed surrogated equation is applicable within the calibrated range of the experimental data, representing asphalt mixtures tested at 20 °C, and its extrapolation beyond this range may lead to reduced accuracy.

### Machine learning model performance

**Fig. 12:**
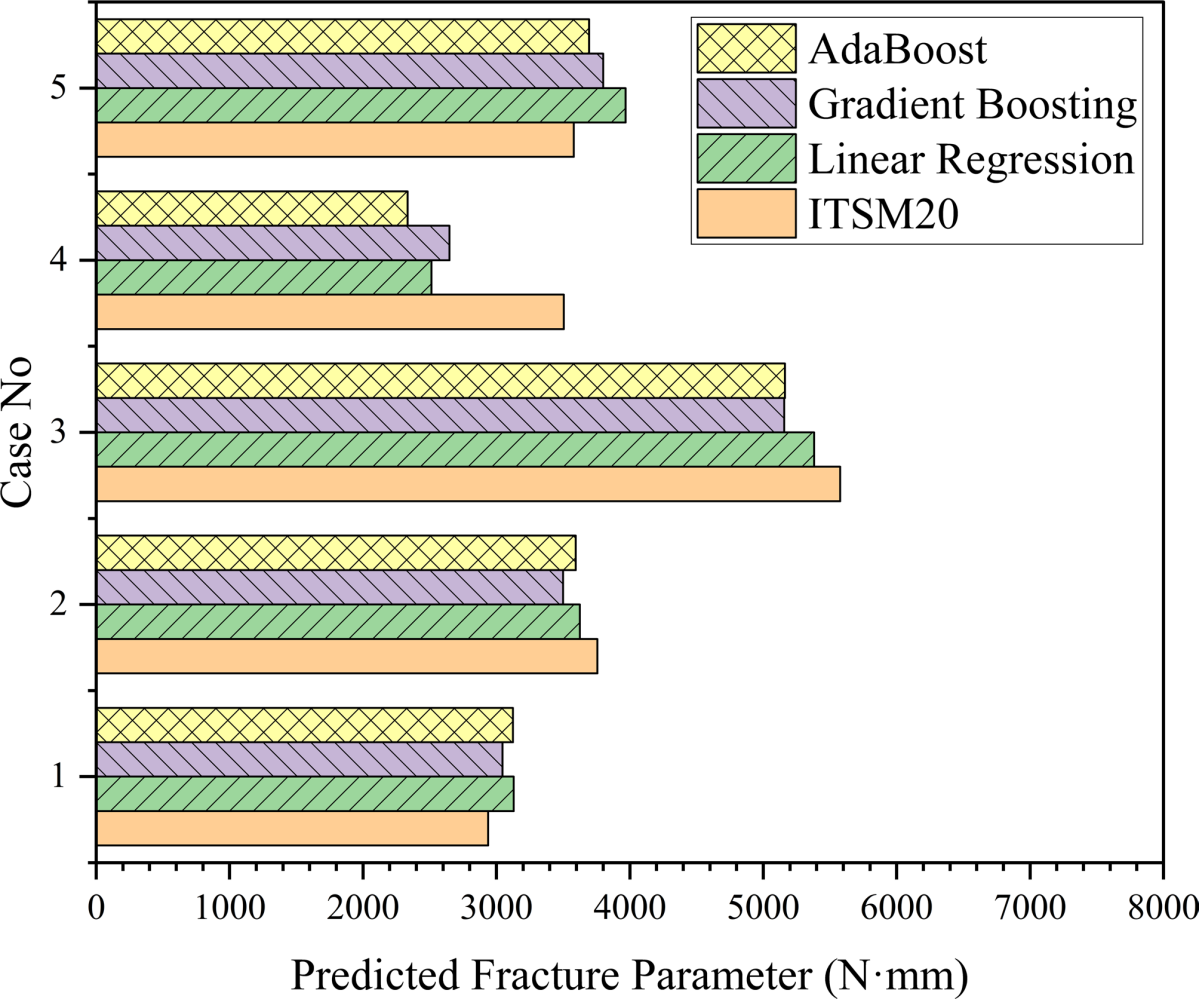
ML model predictions across dataset samples.

The predictive performance of the three machine learning models after correlation analyses (tabulated in Table 4) was evaluated across the dataset using ITSM20 as the targeted feature. Linear Regression tended to overpredict for lower stiffness values and underpredict for mid-range values, resulting in larger deviations for certain samples. AdaBoost exhibited higher variability, particularly underestimating mid-range stiffness cases. In contrast, Gradient Boosting provided more balanced predictions across the full ITSM20 range, closely capturing the trend of the target fracture response while avoiding extreme deviations. The summarization of the comparison of performance is shown in Fig. 12. This consistency suggests that Gradient Boosting is the most reliable model among the three for predicting $\:{G}_{f}$, offering a best framework for subsequent comparisons with experimental and numerical data.

### Integration of physics, experiments, and ML

**Fig. 13:**
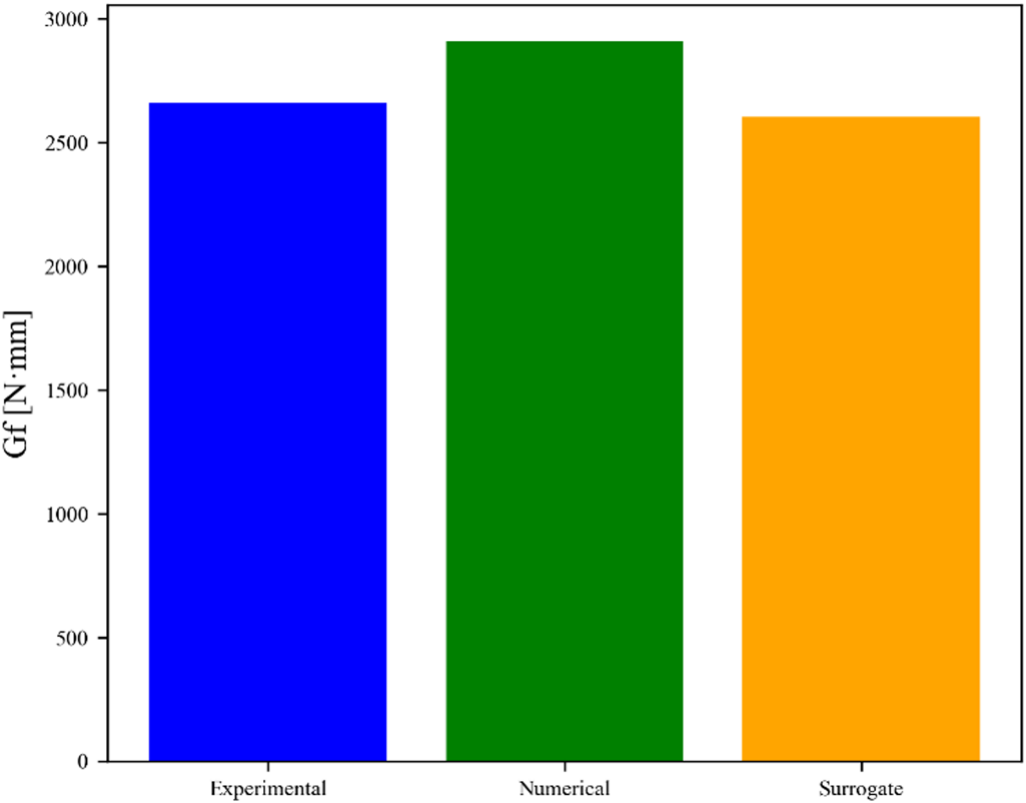
Comparison of all fracture energies.

The present study demonstrates a hybrid and novelty-based approach, applied exclusively to baseline asphalt mixtures (National Highway Authority Class B gradation, 60–70 penetration grade bitumen, no additives) by integrating physical modeling, experimental measurements, and machine learning. The surrogate $\:{G}_{f}$ equation, Eq. 1, was derived based on physical reasoning from the single-edge notch beam concept, incorporating key material properties such as stiffness (ITSM20), mixture stability, and flow characteristics. Experimental fracture tests provided reference energy values (2659.75 N·mm), while FEM simulations offered numerical benchmarks (2910.97 N·mm). The machine learning framework then predicted $\:{G}_{f}$ for each dataset sample using the surrogate equation, capturing trends across the domain of input parameters. Comparison of the mean surrogate Gf (2602.26 N·mm) with experimental and numerical results demonstrates excellent agreement within 2% tolerance, validating the integration of physics-guided modeling with data-driven predictions. A chart in Fig. 13 represents the comparison of all integrated results of $\:{G}_{f}$. This novelty-based approach, adopted in this study, not only ensures dimensional consistency and interpretability but also enables the extrapolation of fracture behavior to untested mixtures, providing a robust framework for performance prediction.

## Conclusion and recommendations

Based on extensive research, a physics-guided surrogate equation was developed to accurately estimate the fracture energy (Gf) of asphalt mixtures using key mixture parameters. Based on extensive research, a physics-guided surrogate equation was developed to accurately estimate the fracture energy (Gf) of baseline asphalt mixtures (National Highway Authority Class B gradation, 60–70 penetration grade bitumen, no additives) using key mixture parameters. The proposed approach was validated through experimental testing and Finite Element Method (FEM) simulations, demonstrating strong agreement between predicted and observed results. Correlation and Machine Learning (ML) analyses confirmed that mixture stability, flow, and stiffness modulus (ITSM20) are the most influential parameters governing the fracture response of asphalt mixtures.

In practical engineering terms, the proposed surrogate model provides a cost-effective and time-efficient tool for estimating fracture energy without requiring exhaustive laboratory testing. This makes it suitable for routine quality control, mixture design optimization, and performance-based pavement evaluation in road construction projects. By predicting fracture resistance at the design stage, engineers can better select bitumen grades, aggregate blends, and binder contents to enhance pavement durability and minimize cracking risks under varying service conditions.

It is recommended that this surrogate fracture approach be further extended to other asphalt mixture types or composite materials exhibiting similar viscoelastic characteristics. Future studies should aim to incorporate temperature- and loading-frequency–dependent effects to improve the model’s generality. Moreover, coupling the surrogate model with advanced ML algorithms and field performance databases could support data-driven pavement management systems, enabling more reliable and sustainable infrastructure design.

## Data Availability

The data presented in this study are available on request from the corresponding author and the data set can also be downloaded from Mendeley website ( [https://data.mendeley.com/datasets/yb4brz3sdx/3](https:/data.mendeley.com/datasets/yb4brz3sdx/3) )

## Abbreviations

AC: Asphalt Content – % (by weight of mix)
CF: Coarse/Fine Ratio
D: Characteristic Beam Dimension (mm)
EffAC: Effective Asphalt Content % (by weight of mix)
FEM: Finite Element Method
Flow: Flow Value (mm)
Gf: Fracture Energy (N mm)
Gmm: Maximum Theoretical Specific Gravity
ITSM20: Indirect Tensile Stiffness Modulus at 20 °C (MPa)
ITSM30: Indirect Tensile Stiffness Modulus at 30 °C (MPa)
K: Scaling Constant
MAE: Mean Absolute Error (Target unit)
MAPE: Mean Absolute Percentage Error (%)
ML: Machine Learning
MSE: Mean Squared Error – (Target unit)²
P4: Percent Retained on Sieve #4 (%)
P34: Percent Retained on 3/4” Sieve (%)
P38: Percent Retained on 3/8” Sieve (%)
P200: Percent Passing Sieve #200 (%)
R²: Coefficient of Determination
RMSE: Root Mean Squared Error (Target unit)
SENB: Single Edge Notch Beam
SFR: Stability to Flow Ratio (Unitless)
Stab: Stability (N)
UWt: Unit Weight (kN/m³)
Va: Air Voids (%)
Vis: Viscosity (Pa s)
